# Profiles of Achievement Motivation and Performance in Middle School: Links to Student Background and Perceived Classroom Climate

**DOI:** 10.3389/fpsyg.2022.820247

**Published:** 2022-05-30

**Authors:** Rasa Erentaitė, Rimantas Vosylis, Daiva Sevalneva, Eglė Melnikė, Saulė Raižienė, Daiva Daukantaitė

**Affiliations:** ^1^Faculty of Social Sciences, Arts, and Humanities, Kaunas University of Technology, Kaunas, Lithuania; ^2^Institute of Psychology, Mykolas Romeris University, Vilnius, Lithuania; ^3^Institute of Psychology, Vilnius University, Vilnius, Lithuania; ^4^Department of Psychology, Lund University, Lund, Sweden

**Keywords:** achievement motivation, academic performance, social-economic-cultural context, classroom climate, latent profile analyses

## Abstract

Achievement motivation and performance at school are reciprocally related, however, empirical studies report a large variability of findings and, in some cases, weaker than expected associations between these constructs. To further our understanding of the motivation–performance link, we examined typical patterns of motivation and performance and their correlates, in two cohorts of 8th-grade students (*N*_1_ = 998, *N*_2_ = 441). As expected, we identified both concordant and discordant patterns of achievement motivation and performance. In two subgroups, specifically, those characterized by low motivation and low performance (34% of the sample) and those characterized by high motivation and high performance (18% of the sample), the levels of motivation were highly concordant with scores on math and reading tests. In contrast, the other two profiles—weak motivation with elevated performance (38% of all sample) and high motivation with low performance (9% of the sample) had divergent patterns of motivation and performance. The subgroups also differed on student socio-economic background, special educational needs, gender, as well as perceptions of classroom climate. Overall, our findings reveal context-dependent patterns of the relationship between aspects of achievement motivation and performance.

## Introduction

Achievement motivation and performance at school are closely interrelated. At any stage of comprehensive schooling, adaptive motivation is considered a critical precursor for a successful academic performance, while higher performance is expected to strengthen students’ achievement motivation (Koenka, 2020; Vu et al., 2021). Indeed, numerous studies over several decades have found positive links between specific aspects of students’ motivation to learn and their academic performance (for meta-analyses and reviews, see Hansford and Hattie, 1982; Valentine et al., 2004; Huang, 2011; Korpershoek et al., 2019; Hattie et al., 2020; Vu et al., 2021).

Despite substantial empirical support for the positive association between aspects of achievement motivation and performance, there is considerable variability in the findings on the strength of the links. In some cases, the motivation–performance links appear surprisingly weak (e.g., Hansford and Hattie, 1982; Valentine et al., 2004; Huang, 2011; Hattie et al., 2020), even though there are some exceptions with single studies showing strong relationships (e.g., Trigueros et al., 2020). Indeed, some studies did not find any substantial association between aspects of achievement motivation and performance at school (e.g., see such studies listed in meta-analyses by Valentine et al., 2004; Korpershoek et al., 2019). Moreover, the findings of several studies challenge the conceptualization of the motivation–achievement link as a linear continuum (Roeser et al., 1999; Korhonen et al., 2014; Parhiala et al., 2018; Widlund et al., 2018). Specifically, some subgroups of students with reduced achievement motivation had no apparent problems in academic performance and vice versa, for some students, low performance was not necessarily accompanied by reduced motivation (Roeser et al., 1999; Korhonen et al., 2014; Parhiala et al., 2018; Widlund et al., 2018). These findings suggest that some non-linear conceptualization of the motivation–performance association may be necessary to better describe the variations in empirical data.

In other words, we need to understand how the relation between achievement motivation and performance at school may play out differently for different students. For some of them, motivation to learn may be substantially linked to their learning results, while for others performance may diverge from their achievement motivation. These interindividual differences may become particularly salient in middle school, when motivation to learn and school performance drop substantially, especially among low performing, anxious, socio-economically vulnerable students (Eccles et al., 1993; Eccles and Roeser, 2009). In order to better address the learning needs of students in middle school years, we need to improve our understanding of the heterogeneity in their achievement motivation and performance. In this study, relying primarily on a person-oriented approach, we aim to identify different patterns of achievement motivation and performance in two cohorts of 8th-grade students. Our study also contributes to a better understanding of student and classroom characteristics related to different profiles of motivation and performance in middle school.

### Achievement Motivation as Competence Beliefs, Values, and Affect

Achievement motivation is what moves students’ choice, persistence, effort, and engagement on achievement-related tasks and activities (Wigfield et al., 2015), including academic tasks and activities at school. Expectancy–value theory (Eccles et al., 1983; Eccles and Wigfield, 2020) provides one of the most established models of achievement motivation, rooted in social-cognitive perspective. The model discusses proximal and distal predictors of achievement-related choices, engagement, and performance. Based on this model, the most critical motivational constructs include students’ expectancies for academic success, centered around the beliefs about their own academic competence, and subjective value that students attribute to academic tasks or activities. The model also stresses the importance of achievement-related affect in determining achievement-related choices and engagement (Eccles and Wang, 2012; Wigfield et al., 2015). These motivational processes (competence beliefs, academic values and affect) appear among the most proximal psychological determinants of students’ choice and effort (e.g., Wang and Eccles, 2011; Eccles and Wang, 2012; Eccles et al., 2015; Wigfield et al., 2015; Eccles and Wigfield, 2020), therefore, we build the construct of achievement motivation around them in this study.

Students tend to engage in academic tasks if they expect to experience success in these tasks (Eccles et al., 1983; Eccles and Wigfield, 2020). Students’ beliefs about their own academic competence are at the center of their expectations for success with academic tasks (Eccles et al., 1983; Eccles and Wigfield, 2020). Beliefs about one’s academic competence may be situational, that is, related to a specific task at a particular time (referred to as task-specific expectancies for success), or more stable, general appraisals of one’s competence in academic activities (referred to as academic self-concepts; Eccles and Wigfield, 2020). Academic self-concept in its broadest sense is individuals’ knowledge and perceptions about themselves in the context of achievement (Bong and Skaalvik, 2003). Although specific operationalizations of academic self-concept vary considerably across studies, the construct generally reflects subjective answers to the question “Can I succeed in this task (or academic tasks in general)?” (Eccles et al., 1993; Eccles and Wigfield, 2020).

The value that a student sees in academic tasks or learning in general reflects a subjective answer to the question “Do I want to succeed in this task (or learning at school)?” (Eccles et al., 1993). As such, academic task value is based on a student’s understanding of the possible personal outcomes of engaging in the academic task. The overall value attributed to academic activities depends on a number of characteristics of these activities, but also on the broader needs, values, goals, and past experiences of a student (Eccles and Wigfield, 1995). Students with high academic task value see academic activities as interesting and pleasurable (referred to as interest–enjoyment aspect of value), important (attainment value), instrumental for achieving their longer-term goals (utility value), and requiring a relatively low investment or sacrifice to succeed (referred to as cost; Wigfield and Eccles, 2002; Eccles and Wigfield, 2020).

Competence and value appraisals are intertwined with the emotional aspects of motivational processes, such as students’ affective reactions to school or academic tasks (Wang and Eccles, 2011; Eccles and Wigfield, 2020). EVT conceptualizes achievement-related emotional reactions, referred to as “affective reactions and memories” (Eccles and Wigfield, 2020), among the most proximal predictors of achievement-related behaviors. Based on EVT, positive affect stems from previous success experiences in achievement-related settings (e.g., school) and tasks (Eccles and Wang, 2012; Eccles et al., 2015). These affective reactions then feed into the perceptions of academic task value, especially, its interest–enjoyment aspect, but also the perceptions of cost (Eccles and Wigfield, 2020). Positive affect gained from pleasurable participation in academic activities “heightens engagement, whereas negative affect reduces it, and increases the cost of activities” (Eccles et al., 2015, p. 671). Thus, the affective processes tie together the critical constructs of EVT—the success-related appraisals, task value, and cost judgments.

The interplay of the motivational processes presented above is at the core of achievement motivation at school, thus, we have operationalized students’ motivation as academic task value, academic self-concept, and school-related affect. We explored the links between achievement motivation and students’ performance from the variable and person-oriented perspectives (Bergman et al., 2003). The need for these complementary methodological approaches in studying the links between achievement motivation and performance at school emerged from the variability of existing findings.

### The Links Between Achievement Motivation and Performance at School

Most contemporary motivational theories consider that achievement motivation and performance at school are inextricably related, but discussions regarding the strength of the association continue (Hattie et al., 2020; Vu et al., 2021). Existing studies report varying sizes of the links between motivational constructs and performance. For the most well-studied aspect of achievement motivation, academic self-concept, the mean longitudinal links with academic performance were characterized as medium to large in Huang’s (2011) meta-analysis. However, the cross-sectional correlations between self-concept and performance reported in this meta-analysis varied between 0.17 and 0.30 (Huang, 2011), which indicates a weak to moderate cross-sectional association. An earlier meta-analysis (Valentine et al., 2004) reported a small average regression coefficient of 0.08 between self-related aspects of motivation (including academic self-concept) and subsequent academic performance, however, the effect varied substantially (between −0.12 and 0.36) across the studies included in this meta-analysis, which is in line with an even earlier meta-analysis on the same issue (Hansford and Hattie, 1982). A cross-sectional correlation between school-related affect and achievement was characterized as weak in Korpershoek et al. (2019) meta-analysis—the average strength of the correlation was 0.18 and depending on the instrument it ranged from 0.07 to 0.42 across the reported studies. While we did not find any meta-analytic studies on the link between academic task value and school performance among secondary school students, a null to weak correlation of task value with knowledge and skills was reported in a meta-analysis with adult learners (Bauer et al., 2015). To summarize, most existing studies provide support for a positive association between motivation aspects and performance at school, however, substantial variations in the findings, as well as skepticism regarding the size of the association remains (Hattie et al., 2020).

Indeed, some studies did not find any substantial association between the aforementioned aspects of achievement motivation and academic performance (e.g., see such studies listed in meta-analyses by Valentine et al., 2004; Korpershoek et al., 2019). Such findings encourage researchers to seek explanations for substantial variations across different studies and samples. Several moderators of the motivation–performance link, including the breadth and congruence of the academic domains in focus (Valentine et al., 2004; Huang, 2011), the operationalization and measurement of both motivation and performance aspects (Hansford and Hattie, 1982; Korpershoek et al., 2019), as well as student socio-economic status, grade, ability (Hansford and Hattie, 1982), and a few other moderators help to partly explain the observed variations in motivation–performance link. In addition, external influences may explain why the role of internal motivational processes is in some cases weaker than expected for academic performance (Vu et al., 2021). Such external influences include rewards and requirements (e.g., deadlines or exams), the quality and extent of support for learning (e.g., the quality of teaching and study materials), distorted perceptions of one’s own performance (e.g., due to social comparison or feedback). Under the influence of these external factors, strong academic performance may not necessarily lead to a higher motivation, while poor actual performance may not always be detrimental for a willingness to learn (Vu et al., 2021).

The effects of moderators and external factors suggest that the links between motivation aspects and performance at school are not always positive or linear. In other words, a tendency for stronger motivation to be related to higher performance may not apply to all students or to all situations. Despite this, most previous studies examining motivation–performance links rely predominantly on a linear, variable-oriented analytical approaches, which assume a homogeneity of association across the whole population and apply aggregate estimates of association to describe the functioning of all students (Parhiala et al., 2018; Linnenbrink-Garcia and Wormington, 2019). While such an approach is important for understanding the unique associations between aspects of achievement motivation and performance, it has theoretical and methodological limitations for identifying and understanding the heterogeneity of this link across the student population, as well as for explaining a null or weaker than expected aggregate association.

With this in mind, we aim to explore the potential heterogeneity of the motivation–performance links by applying a person-oriented approach (Bergman et al., 2003) in our study. This approach enables us to identify student subgroups with different patterns of motivation and performance without assuming a uniform association between these constructs across the whole sample. It also accounts for the interplay between different aspects of motivation, which most often remains unaccounted for in variable-oriented studies (Valentine et al., 2004). Following a person-oriented perspective, we simultaneously examine three key aspects of achievement motivation: academic self-concept, academic task value, and positive school-related affect, and two aspects of school performance—standardized test scores in reading and math.

### Profiles of Achievement Motivation and Performance and Their Correlates in Middle and High School

A few previous person-oriented studies have addressed the potential heterogeneity in students’ achievement-related functioning among middle school students by identifying distinct profiles characterized by combinations of motivational, emotional, and performance variables (Roeser et al., 1999; Korhonen et al., 2014; Parhiala et al., 2018; Widlund et al., 2018). Not surprisingly, the findings of these studies show that generally, student profiles with high motivation performed better than those with low motivation (Roeser et al., 1999; Korhonen et al., 2014; Parhiala et al., 2018). However, some less expected outcomes also challenge the conceptualization of motivation–achievement link solely as a linear continuum. Specifically, a subgroup of students characterized by *poor academic value* profile, identified at both 7th and 8th grade in the US (Roeser et al., 1999), did not show worse academic performance (year-end grade point average, school failure) compared to profiles marked by very high scores on academic value. Substantially lower performance was only observed among students characterized by the *multiple risks* profile, in which low academic value was accompanied by poor emotional functioning (Roeser et al., 1999).

Similar observations can be made from the results of person-oriented studies with lower secondary school students in Finland. Among 9th-grade students (Parhiala et al., 2018), a *low wellbeing* profile subgroup with an average motivation and poor emotional functioning did not show reduced performance in math or reading (Parhiala et al., 2018). Moreover, these students had the highest scores in reading fluency compared to other subgroups. Another subgroup characterized by an *average motivation*/*average wellbeing* profile did not perform substantially worse than the *high motivation*/*high wellbeing* subgroup on reading fluency and comprehension (Parhiala et al., 2018). Both subgroups characterized by average motivation had higher than expected chances of facing no performance difficulties in reading and math (Parhiala et al., 2018). Another study in Finland (Widlund et al., 2018) revealed a *low-performing* profile of 9^th^-grade students with very low math performance, but average motivation and no indications of emotional alienation from school. Surprisingly, despite their low achievement, these students seemed to do well in their motivation and relationship with school. In contrast, Korhonen et al. (2014) identified a subgroup of 9th-grade students with a *low academic wellbeing* profile who performed average in reading, math, and spelling, but their motivational functioning and relationship to school were very poor—they had the lowest score in academic self-concept, the highest perceived learning difficulties and the highest emotional alienation from school across all identified subgroups.

Taken together, the findings of these person-oriented studies reveal that among some students, reduced achievement motivation is not accompanied by problems in academic performance, and vice versa, low performance does not necessarily mean reduced motivation and poor emotional functioning at school. This shows that a linear conceptualization of the motivation–performance link is insufficient to describe the actual variations in empirical data. However, many questions remain after looking at the existing findings from person-oriented studies: Is average motivation enough to sustain high levels of performance? Which combinations of motivational variables are associated with low performance? Do the profiles of academic functioning identified in the Finnish and US samples also characterize students in countries with different educational systems/levels of academic performance (It is important to consider that the countries represented in these existing studies have generally high performance, e.g., OECD, 2019)? These questions require further exploration of the heterogeneity in academic performance and motivation from a person-oriented perspective.

Moreover, it is essential to understand the correlates of different patterns of academic performance and motivation. Rather little is known about the students who comprise these identified profiles. Most existing person-oriented studies only looked at the gender composition of identified subgroups, with very scarce findings on socio-economic background of students within these specific patterns of motivation and performance. There is also a lack of understanding about the classroom environment characteristics related to these different profiles. Our study aims to address this gap by looking at a range of student background characteristics and the students’ perceptions of classroom environment across different profiles of students.

### The Current Study

In the present study, we aim to identify the patterns achievement motivation and performance at school in two cohorts of 8th-grade students. Based on the existing findings, we expect to find concordant profiles in which motivation and performance levels are similar (i.e., all high, average, or low). In previous studies, large profiles with high performance/high motivation and average performance/average motivation were reported (Korhonen et al., 2014; Widlund et al., 2018). In addition to concordant profiles, we also expect to find subgroups with mixed patterns of motivation and performance or discordant profiles. As in previous person-oriented studies (Roeser et al., 1999; Korhonen et al., 2014; Parhiala et al., 2018; Widlund et al., 2018), we expect some students to show high performance (i.e., get higher than average test scores) despite reduced motivation or have low levels of performance (i.e., get lower than average test scores) while not showing poor motivation patterns.

Our second aim is to identify student background characteristics that may be related to different profiles of academic motivation and performance. We include a broader list of student socio-demographic characteristics compared to previous person-oriented studies. Specifically, we analyze profile composition with respect to student gender, socio-economic background, special educational needs, and school location. We expect students in profiles with higher motivation and performance to come from more favorable social backgrounds (i.e., from families with higher SEC), and students in profiles with lower motivation and performance to come from less favorable social backgrounds (i.e., from families with lower SEC; based on Roeser et al., 1999); we also expect profiles with poor functioning to have more male students (based on Roeser et al., 1999; Korhonen et al., 2014; Parhiala et al., 2018; Widlund et al., 2018).

Our study also contributes to understanding school-related correlates of motivation and performance profiles among 8th-grade students. Specifically, our third aim is to analyze how students characterized by different profiles perceive important characteristics of classroom environment. From the developmental motivational perspective (Roeser et al., 1998; Roeser and Galloway, 2002; Eccles and Roeser, 2009; Wigfield et al., 2015), a stronger motivation to learn and better academic performance can be achieved by providing opportunities for adolescents at school to experience “fit” between their classroom environment and their salient developmental needs. Based on this perspective, instructional and interpersonal processes in a classroom, referred to as classroom climate, should promote adolescents’ developmental needs associated with competence development, experience of autonomy, and supportive relationships (Eccles and Roeser, 2009). The fit between classroom climate and these developmental needs should enhance adolescent students’ motivation and performance (Roeser et al., 1998; Roeser and Galloway, 2002; Eccles and Roeser, 2009). In contrast, aspects of classroom life that mismatch with these needs should produce academic, emotional, and behavioral alienation from school and learning (Roeser et al., 1998; Roeser and Galloway, 2002). We hypothesize that students who perceive classroom climate as supportive of their developmental needs will be characterized by profiles with high levels of motivation and performance.

Our study also contributes to expanding the educational contexts in which motivation and performance patterns are analyzed. Both samples used in our study come from the Eastern Europe, from the Lithuanian educational context. The results of national assessments and international surveys of educational achievement in Lithuania reveal a number of challenges with regards to the academic performance of middle school students. Specifically, recent rounds of PISA assessments show that Lithuanian 15-year-olds (8th-9th-grade students) perform below the average of Organisation for Economic Co-operation and Development (OECD) countries (OECD, 2013, 2016, 2019). National Examination Center (NEC) assessments and surveys show that there is a substantial share of low achieving students and students with learning difficulties (NEC, 2015, 2018). Moreover, wide student achievement gaps by gender, region (urban/rural), student socio-economic background, and school have been documented (NEC, 2018; OECD, 2019). In this challenging educational context, it is particularly important to understand the patterns of motivation and performance among middle school students and to compare them with previous findings from countries with much higher levels and smaller gaps of student achievement. The school network, curriculum, and national assessment structure makes the 8th grade an important turning point in the educational path of the Lithuanian students, therefore, we focused on two cohorts of 8th-grade students.

## Materials and Methods

### Sample

For this study, we used open-access data from the National Survey of Student Achievement (NSSA) in Lithuania. NSSAs are a series of national educational studies conducted from 2002 to 2016, designed to provide countrywide information about student achievement and educational context. The NSSAs include standardized student achievement tests in math, reading, and other subjects, and self-report questionnaires on student background, motivation, and learning environment at home and school. The NSSAs are based on nationally representative stratified two-stage nested samples. The students are sampled by randomly selecting schools and then randomly selecting a class/classes from selected schools.

The present study uses data from the NSSA rounds of 2012 and 2015. Only students from schools with Lithuanian as the language of instruction and only those who completed the Mathematics and Reading tests were included in the current analyses. The study sample for 2012 includes 998 students (50.1% female, from 160 comprehensive schools/212 classes, 6.4% had special educational needs, 13.4% from rural locations). The sample for 2015 includes 441 students (48.8% female, from 147 comprehensive schools/166 classes, 6.1% had special educational needs, 15.6% from rural locations). The sample proportions by urban population, gender, special educational needs, school types correspond to the national distribution. Both rounds of NSSAs were administered in schools at the end of a school year. Student testing (standardized tests and questionnaires) took about 150 min.

#### Ethical Considerations

The NSSA data were collected by the national authorities according to the national legal regulations of Lithuania (Regulations for Conducting Studies of Student Achievement, 2008), which include provisions on ethics and confidentiality of the study participants. The data are currently available from the National Agency for Education.^1^

### Measures

#### Achievement Motivation

Student self-report scales were used to measure three aspects of achievement motivation: academic task value, academic self-concept, and positive school-related affect. The list of items for each motivation aspect are presented in Annex 1. Students were asked to rate each statement using a four-point Likert-type scale ranging from 1 (strongly disagree) to 4 (strongly agree). Following Eccles’ works on task value (e.g., Eccles and Wigfield, 1995, 2020), academic task value was defined as the subjective value of studying in general and was measured by five items assessing students’ perceived interest in and importance of studying. Further, following Eccles’ works (e.g., Eccles and Wigfield, 1995, 2020), four items reflecting individuals’ self-beliefs about their ability to perform learning activities and succeed in academic domain even facing a challenging task were used to measure students’ academic self-concept. Finally, we used four items reflecting students’ affective reactions, such as safety, enjoyment, and excitement, toward school and classroom to assess school-related affect. Similar items reflecting students’ level of enjoyment, liking, and safety at school were used to assess emotional school appraisals in previous studies (e.g., Wang and Eccles, 2011). Composite scores for each aspect of achievement motivation were created by computing the mean of the items that make up the motivation scales. In the study, all three academic motivation scales showed good internal consistency as Cronbach’s alphas were between 0.69 and 0.77 in 2012 and between 0.76 and 0.79 in 2015.

#### Academic Performance

Each student received standardized tests in two subject areas: math and reading (Lithuanian). The mathematics test targets mathematics skills in five content domains: basic numbers and calculations, algebra, geometry, data and probability, and problem-solving. Aggregate scores obtained from different math domains show very high consistency—Cronbach’s alphas were between 0.86 and 0.92 in 2012 and 0.92–0.93 in 2015. The reading test targets reading skills in four content domains: retrieval of explicitly stated information, inference making, analysis, and interpretation and evaluation. Aggregate scores obtained from different reading domains show good reliabilities—Cronbach’s alpha varied between 0.76 and 0.85 in 2012 and 0.85–0.86 in 2015. The aggregate scores for both math and reading are standardized so that the mean of the score in each assessment is 500 and the SD is 100.

#### Social–Economic–Cultural Background

We utilized a composite index for SEC consisting of multiple student self-report items specifying: a number of books at home (measured on a 5-point ordinal scale); possession of six types of things indicating family wealth, cultural and educational resources: own books, encyclopedia, musical instrument, works of art/albums, three or more computers, and dishwasher at home (measured on a binary scale *yes*/*no*); eligibility of free meals at school (measured on a binary scale *yes*/*no*); frequency of consultation by a private tutor (measured on a binary scale 0 = *never*/*almost never* and 1 = *sometimes*/*at least once a month*). Large cross-national studies on adolescent educational achievement and health include similar measures of SEC background (e.g., Currie et al., 2008; OECD, 2014). We used a confirmatory factor analysis (CFA) to construct the SEC index on both datasets separately. In particular, we employed weighted least squares mean and variance adjusted (WLSMV) estimation implemented in the *Mplus 8.6* software to account for the categorical (in most cases binary) nature of the data (Brown, 2015). Results of CFA indicated that an assumed single factor structure for SEC index had an acceptable fit with data in each year (*χ*^2^ = 70.165, *df* = 25, *p* < 0.001, CFI = 0.935, TLI = 0.935, RMSEA = 0.043 [90% CI: 0.031–0.055], SRMR = 0.059 and *χ*^2^ = 47.147, *df* = 25, *p* = 0.005, CFI = 0.947, TLI = 0.924, RMSEA = 0.046 [90% CI: 0.025–0.065], SRMR = 0.070, respectively in 2012 and 2015).

In the light of these findings, we also performed the analysis of measurement invariance (MI) of the SEC scale to assess the equivalence of factor loadings across the two datasets. Results of a configural invariance multiple-group CFA model had an acceptable fit with data (*χ*^2^ = 122.814, *df* = 52, *p* < 0.001, CFI = 0.936, TLI = 0.911, RMSEA = 0.044 [90% CI: 0.034–0.054], SRMR = 0.063) and so did the metric invariance model (*χ*^2^ = 122.933, *df* = 61, *p* < 0.001, CFI = 0.944, TLI = 0.934, RMSEA = 0.038 [90% CI: 0.028–0.048], SRMR = 0.067). A comparison of the two models also indicated that the metric invariance model, in which factor loadings were constrained to be equal across groups, was not statistically significantly different from the configural invariance model, in which factor loadings were estimated freely in each group (Δ*χ*^2^ = 11.599, Δ*df* = 9, *p* = 0.237). In conclusion, the same factor structure was retained across different datasets. Factor scores resulting from the performed metric invariance CFA model were saved and used as the estimates of the SEC index. Reliability of the composite was also sufficient and similar for each year: *ρ* = 0.69 for 2012 and *ρ* = 0.70 for 2015. Higher scores indicate higher SEC.

#### Other Socio-Demographic Characteristics

Students provided information about their gender measured on a binary scale *male/female*. Location was measured on a binary scale *urban/rural*. Specialized program was also indicated using a binary scale asking whether school curriculum was adapted to a student’s special learning needs (*learning difficulties*, yes/no). Information about location and special learning needs was taken from official school records.

#### Classroom Climate

Aligned with EVT (Eccles and Roeser, 2009; Wigfield et al., 2015), 14 items were used to measure students’ perception of three dimensions of classroom climate: teacher–student relationship, classroom management, and motivational climate (see Annex 1). Responses were provided on a four-point Likert-type scale ranging from 1 (strongly disagree) to 4 (strongly agree). For assessing teacher–student relationship, we used four items reflecting a student’s perception about the emotionally supportive interaction with teachers. When teachers are sensitive, trustful, and are respectful of students, they create the contexts that support positive development (Eccles and Roeser, 2009). Classroom management is the characteristic of instruction that provides students with a sense of learning process predictability and enhance students’ academic motivation (Eccles and Roeser, 2009). To measure classroom management we used seven items capturing different teaching practices that allow creating a well-structured and predictable studying environment. And finally, we used three items to measure one more aspect of instruction—motivational climate. These three items reflect student’s perspective on teachers’ provision of support for their intrinsic interest (Wigfield et al., 2015). Composite scores for each dimension of classroom climate were created by computing the mean of the items that make up each scale. In the study, all three scales of classroom climate showed good internal consistency as Cronbach’s alpha reliabilities were between 0.76 and 0.82 in 2012 and between 0.79 and 0.85 in 2015.

### Data Analysis

First, we inspected the correlations between study variables. Cohen’s (1988) conventions were used to interpret the size of the correlations. A correlation coefficient in the range of 0.10–0.29 indicates a weak correlation; a coefficient in the range of 0.30–0.49 indicates a moderate correlation; a coefficient of 0.50 or larger represents a strong correlation.

Second, Latent Profile Analyses (LPA) were conducted to uncover academic achievement and motivation profiles, using the Maximum Likelihood Robust (MLR) in *Mplus 8.5*. Scores on the academic task value, academic self-concept, positive school-related affect, math standardized test, and reading standardized test were used as profile indicators. All scores were standardized separately for each study dataset before the analysis. To ensure that the log-likelihood value is replicated and that it does not represent local maxima, all models were estimated using 10,000 random sets of start values with 500 iterations.

Using the 2012 dataset, a set of models containing from one to eight latent profiles were tested and compared against each other in terms of fit, and then the same analysis was repeated for 2015. In both cases, the best-fitting model was chosen by investigating a set of criteria suggested by Masyn-Garcia (2013). First, we looked for smaller values in Bayesian information criterion (BIC), approximate weight of evidence (AWE), and consistent Akaike’s information (CAIC) criterions. In addition, we inspected which solutions were characterized by higher entropy values, as these would indicate a better model fit. Lastly, we looked for significant value of *ps* of the Lo–Mendell–Rubin likelihood ratio test (LMR-LRT), which would indicate that the model with *k* classes fitted the data better than the model with *k*-1 classes.

Third, and once the best-fitting LPA model was selected for the two datasets, we conducted a latent profile similarity analysis. Specifically, using a stepwise approach and guidelines provided by Morin et al. (2016), we tested for configural (number of profiles), structural (within-profile means), dispersion (within-profile variability), distributional (proportion) similarity of the profiles uncovered in 2012 and 2015 study datasets. A configural similarity model is a multiple-group latent profile model that estimates a set of profiles in two groups and does not impose any cross-group parameter constraints. A structural similarity model is the same as the configural similarity model but includes cross-group equality constraints on within-profile means. A dispersion similarity model is the same as the structural similarity model but includes cross-group equality constraints on within-profile variance estimates. Lastly, the distributional similarity model is the same as the dispersion similarity model but includes cross-group equality constraints on class probabilities. To estimate if the included model constraints worsen model-data fit, we checked if BIC, CAIC, and AWE values increased compared to the model estimated in the previous step. In our case, all three values decreased, and we therefore concluded that a certain type of similarity holds.

As the last step of our analysis, we investigated how demographic characteristics, such as gender, SEC, location (rural vs. urban), specialized teaching program, and three aspects of classroom climate predict profile membership. To conduct this analysis, we saved the most likely class membership for each study participant and used this variable as an outcome in a multinomial logistic regression performed with SPSS 25. We switched the reference group several times during the analysis to obtain all possible comparisons. The magnitude of the predictor effects (odds ratios) on profile membership were interpreted by using the guidelines provided by Chen et al. (2010). They suggest that odds ratios higher than 1.68 (or lower than 0.60) indicate a weak effect (association), odds ratios higher than 3.47 (or lower than 0.29) indicate a mediocre effect, and odds ratios higher than 6.71 (or lower 0.15) indicate a strong effect.

## Results

### Correlations and Descriptive Statistics for Study Variables

Table 1 presents the correlations, means, and SDs for study variables. Regarding the size and statistical significance of correlations between study variables, the results were similar across the 2012 and the 2015 datasets. As such, we address them together.

**Table 1:** Means (M), SD, and correlations between the study variables.

	1	2	3	4	5	6	7	8	9	10	11	12
SEC (cont.; normalized)		−0.04	−0.25^*^	−0.08^*^	0.30^*^	0.30^*^	0.16^*^	0.13^*^	0.01	−0.03	−0.03	0.02
Specialized program (binary)	−0.22^*^		0.04	0.02	−0.16^*^	−0.10^*^	0.05	0.07^*^	0.08^*^	0.09^*^	0.03	0.05
Location (binary; rural)	−0.19^*^	0.13^*^		−0.04	−0.15^*^	−0.15^*^	0.01	0.03	0.06	0.09^*^	0.07^*^	0.05
Gender (binary; male)	−0.04	0.07	0.02		0.02	−0.26^*^	−0.13^*^	0.10^*^	−0.13^*^	0.01	0.00	−0.04
Math test score	0.42^*^	−0.32^*^	−0.20^*^	−0.01		0.59^*^	0.16^*^	0.15^*^	0.06^*^	−0.07^*^	−0.08^*^	−0.07^*^
Reading test score	0.40^*^	−0.35^*^	−0.15^*^	−0.31^*^	0.66^*^		0.16^*^	0.04	0.09^*^	−0.13^*^	−0.11^*^	−0.11^*^
Academic task value	0.23^*^	−0.07	0.06	−0.11^*^	0.17^*^	0.24^*^		0.55^*^	0.24^*^	0.29^*^	0.27^*^	0.29^*^
Academic self-concept	0.06	0.03	0.11^*^	0.09	0.10^*^	0.09	0.53^*^		0.26^*^	0.29^*^	0.29^*^	0.29^*^
School-related affect	0.07	−0.06	0.07	−0.06	0.07	0.19^*^	0.42^*^	0.34^*^		0.31^*^	0.34^*^	0.32^*^
Motivational climate	−0.09	0.11^*^	0.16^*^	0.06	−0.13^*^	−0.10^*^	0.34^*^	0.43^*^	0.41^*^		0.75^*^	0.77^*^
Classroom management	−0.08	0.07	0.15^*^	0.03	−0.07	−0.06	0.32^*^	0.41^*^	0.41^*^	0.76^*^		0.80^*^
Teacher-student relationship	−0.03	0.06	0.15^*^	0.01	−0.09	−0.06	0.39^*^	0.45^*^	0.43^*^	0.80^*^	0.79^*^	
M (2012)	0.53	0.06	0.13	0.50	506.03	516.37	3.15	2.77	2.77	2.86	2.81	2.87
SD (2012)	0.19	0.24	0.34	0.50	102.45	74.22	0.43	0.50	0.54	0.59	0.57	0.47
M (2015)	0.57	0.06	0.16	0.52	515.45	498.66	3.16	2.78	2.89	2.93	2.90	2.92
SD (2015)	0.22	0.24	0.36	0.50	101.11	92.06	0.46	0.56	0.57	0.62	0.55	0.48

Correlations for the 2012 dataset are presented above the diagonal, while correlations for the 2015 dataset are presented below the diagonal. SEC, socio-economic and cultural background and Cont., continuous variable. *p < 0.05.

SEC background was significantly yet weakly (Cohen, 1988), positively related to academic task value and academic self-concept. SEC background was also moderately positively related to both math and reading test scores. Academic task value, academic self-concept, and school-related affect scores were weakly related to math and reading test scores, although, in some instances, the correlations were non-significant. All three aspects of classroom climate (motivational climate, classroom management, and teacher–student relationship) were moderately positively correlated with the aspects of students’ motivation (academic task value, academic self-concept, and school-related affect). Interestingly, the classroom climate dimensions were weakly yet significantly negatively related to math and reading test scores. Correlations between the three motivation aspects were moderate-to-strong and positive, while the correlations between the three classroom climate dimensions were positive and strong.

Gender was weakly negatively related to reading test scores, suggesting that male students scored slightly lower on reading. Gender was weakly positively related to academic self-concept and weakly negatively with school-related affect and academic task value, although most of these correlations were only significant for the 2012 dataset. Being in a specialized program and living in a rural area was related to slightly lower achievement scores.

### Latent Profile Analysis

Even though the evidence was slightly ambiguous, the LPA results for the 2012 study dataset favored a four-class model (Table 2). Supporting the four-profile solution, the AWE reached a minimum at the four-profile model. The CAIC and BIC values kept decreasing with models characterized by a higher count of latent profiles. However, there was a visible elbow around the four- and five-profile models for both of these indices. The LMR-LRT test was statistically significant for the two- and four-profile solutions. The entropy for the four-profile model suggested a mediocre classification quality; however, for the 2012 study dataset, neither of the LPA solutions reached the desired (0.80) entropy levels.

**Table 2:** Results of the latent profile analyses.

Model fit indices	1 Profile	2 Profiles	3 Profiles	4 Profiles	5 Profiles	6 Profiles	7 Profiles	8 Profiles
	2012 dataset (N = 982)							
Log-likelihood	−6966.99	−6752.23	−6666.57	−6596.59	−6546.83	−6514.70	−6490.26	−6468.17
Scaling factor	1.44	1.69	1.69	1.54	1.52	1.58	1.50	1.55
Number of parameters	10	16	22	28	34	40	46	52
BIC	14,003	13,615	13,485	13,386	13,328	13,305	13,297	13,295
AWE	14,102	13,773	13,702	13,663	13,664	13,701	13,752	13,809
CAIC	14,013	13,631	13,507	13,414	13,362	13,345	13,343	13,347
Entropy	−	0.73	0.64	0.64	0.71	0.71	0.73	0.74
LMR-LRT test value	−	419.38	167.27	136.66	97.16	62.75	47.73	43.12
LMR-LRT value of *p*	−	<0.001	0.139	0.040	0.173	0.466	0.350	0.532
**Model fit indices**	**1 Profile**	**2 Profiles**	**3 Profiles**	**4 Profiles**	**5 Profiles**	**6 Profiles**	**7 Profiles**	**8 Profiles**
	**2015 dataset (N = 426)**							
Log-likelihood	−3022.34	−2891.40	−2821.85	−2775.99	−2751.89	−2735.77	−2720.85	−2710.13
Scaling factor	1.15	1.28	1.32	1.25	1.34	1.28	1.19	1.23
Number of parameters	10	16	22	28	34	40	46	52
BIC	6,105	5,880	5,777	5,722	5,710	5,714	5,720	5,735
AWE	6,196	6,025	5,976	5,975	6,017	6,076	6,137	6,206
CAIC	6,115	5,896	5,799	5,750	5,744	5,754	5,766	5,787
Entropy	-	0.76	0.74	0.79	0.82	0.81	0.82	0.79
LMR-LRT test value	-	254.86	135.39	89.00	46.90	31.38	29.05	20.85
LMR-LRT value of *p*	-	<0.001	0.015	0.002	0.302	0.466	0.204	0.553
**Model fit indices**	**Analysis of the similarity of the four-profile solution (combined dataset; N = 1,408)**							
	**Configural**		**Structural**		**Dispersion**		**Distributional**	
Log-likelihood	−10198.84		−10251.29		−10262.72		−10263.50	
Scaling factor	1.46		1.50		1.54		1.51	
Number of parameters	57		37		32		29	
BIC	20,811		20,771		20,757		20,737	
AWE	21,395		21,150		21,085		21,017	
CAIC	20,868		20,808		20,789		20,758	
Entropy	0.79		0.81		0.81		0.81	

The evidence advocating for the four-profile solution in the 2015 study dataset was unequivocal. Again, AWE reached its minimum at the four-profile model. The CAIC and BIC were lowest at the five-profile model; however, the differences in CAIC and BIC estimates were minimal for the four- and five-profile solutions. The LMR-LRT test was statistically significant (*p* < 0.05) for the two-, three-, and four-profile models, clearly advocating for the four-profile solution. Importantly, levels of entropy for the four-profile model suggested good classification quality.

Evidence advocating the four-profile model across the two studies suggested that configural similarity holds for the samples. Considering these findings, we built a multiple-group LPA model and proceeded to test the structural, dispersion, and distributional similarity of the four profiles uncovered in two studies. The inclusion of cross-group equality constraints for the within-profile means did not worsen model fit, and the same results were obtained when within-profile variance parameters and class probabilities were constrained to be equal across the two samples. The BIC, AWE, and CAIC indices decreased at each step, suggesting that the included constraints resulted in more parsimonious models. Overall, these results suggested that the four profiles across two samples were similar in terms of their mean and variance levels as well as in their distribution across the two studies.

**Figure 1:**
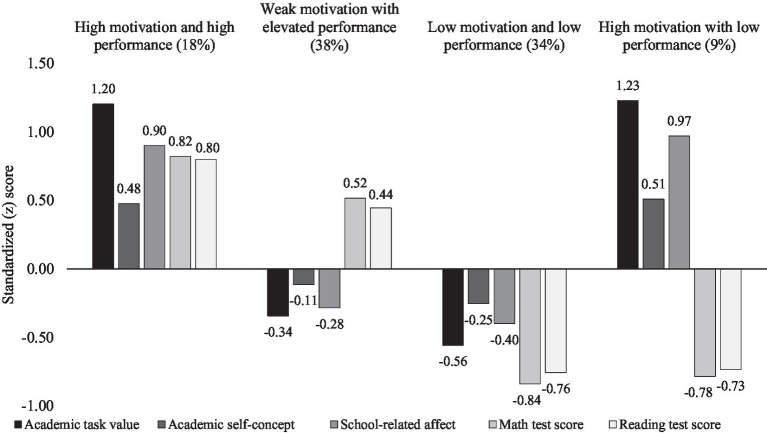
Mean levels of school performance and the three aspects of motivation across the four profiles.

Figure 1 presents the mean levels of the four profiles (the mean levels are identical in the two samples). The first profile was characterized by high scores on math and reading tests and high scores on the three motivational aspects (i.e., academic task value, academic self-concept, and school-related affect). This profile was the second smallest, consisting of slightly less than a fifth of the total sample. We labeled this profile as *high motivation and high performance* (HM/HP). The second profile was the largest, consisting of over a third of the study participants. It was characterized by higher than average math and reading test scores and slightly lower than average motivation scores. We labeled this profile as *weak motivation with elevated performance* (WM/EP). The third profile was slightly smaller but similar in size to the second (WM/EP). The third profile was characterized by low scores on academic tests and motivation; hence, we labeled it as *low motivation and low performance* (LM/LP). Lastly, the fourth profile was the smallest and was characterized by low test scores but surprisingly relatively high motivation. In fact, levels of motivation were similar to the first profile. We labeled this profile as *high motivation with low performance* (HM/LP).

### Predictors of Academic Achievement and Motivation Profiles

In the final step of the analysis, we performed a multinomial logistic regression to examine the relationship between the background predictor variables and membership in the four-profile groups. First, using a stepwise approach, we entered seven predictors (gender, SEC, school location, specialized teaching program, and three aspects of school climate (i.e., motivational climate, classroom management, and teacher–student relationship) into the model and tested which of these improved the model fit. However, log-likelihood difference tests indicated that two predictors (school location and teacher–student relationship) did not improve model-data fit and thus were subsequently removed from model. The log-likelihood difference tests for the remaining five predictor variables were statistically significant (at *p* < 0.05) and suggested that these improved model-data fit. The fit of final overall model including five remaining predictors was: Nagelkerke *R*^2^ = 0.26; *χ*^2^ = 387.03; *df* = 15; *p* < 0.001).

As shown in Table 3, membership in the WM/EP profile, as compared to the HM/HP profile, was significantly (although very weakly) associated with being male, strongly associated with being from a lower SEC background, and moderately associated with lower levels of motivational climate and classroom management. Similarly, belonging to the LM/LP profile, as compared to the HM/HP profile, was weakly associated with being male, strongly associated with a lower SEC background, and moderately associated with lower levels of motivational climate and classroom management. However, in this case, belonging to this profile was also moderately associated with being in a specialized learning program. Belonging to the HM/LP profile as compared to being in the HM/HP profile, was only associated with two variables: It was strongly associated with a lower SEC background and moderately associated with being in a specialized learning program. Being in the LM/LP profile compared the WM/EP profile, was strongly associated with a lower SEC background and moderately associated with being in a specialized program. Being in the HM/LP profile compared to the WM/EP profile, was weakly associated with a lower SEC background, moderately associated with being in a specialized learning program, and weakly associated with high motivational climate and classroom management. Lastly, being in the HM/LP profile compared to the LM/LP profile, was strongly associated with a higher SEC background and weakly associated with high motivational climate and classroom management.

**Table 3:** Multinomial logistic regression analysis results: parameter estimates for the final model.

Comparison	Predictor	*B*	Wald	*p*	Exp(B)	95% CI LB	95% CI UB
Reference: HM/HP Target: WM/EP	Gender (male)	0.45	7.56	0.006	1.56	1.14	2.15
	SEC	−1.74	14.92	<0.001	0.18	0.07	0.43
	Specialized program	−0.32	0.45	0.502	0.73	0.29	1.85
	Motivational climate	−0.89	17.44	<0.001	0.41	0.27	0.63
	Class management	−0.62	7.85	0.005	0.54	0.35	0.83
Reference: HM/HP Target: LM/LP	Gender (male)	0.56	10.50	0.001	1.74	1.25	2.44
	SEC	−5.11	112.67	<0.001	0.01	0.00	0.02
	Specialized program	1.08	6.78	0.009	2.95	1.31	6.66
	Motivational climate	−0.63	7.92	0.005	0.53	0.34	0.83
	Class management	−0.53	5.17	0.023	0.59	0.37	0.93
Reference: HM/HP Target: HM/LP	Gender (male)	0.41	3.10	0.078	1.51	0.96	2.39
	SEC	−2.92	20.96	<0.001	0.05	0.02	0.19
	Specialized program	1.55	11.71	0.001	4.69	1.94	11.37
	Motivational climate	0.25	0.63	0.429	1.29	0.69	2.40
	Class management	0.35	1.16	0.282	1.42	0.75	2.70
Reference: WM/EP Target: LM/LP	Gender (male)	0.11	0.68	0.410	1.12	0.86	1.45
	SEC	−3.37	88.70	<0.001	0.03	0.02	0.07
	Specialized program	1.40	16.73	<0.001	4.07	2.08	7.96
	Motivational climate	0.26	2.37	0.124	1.29	0.93	1.79
	Class management	0.09	0.24	0.623	1.09	0.77	1.55
Reference: WM/EP Target: HM/LP	Gender (male)	−0.04	0.03	0.871	0.97	0.63	1.48
	SEC	−1.19	4.18	0.041	0.31	0.10	0.95
	Specialized program	1.87	20.50	<0.001	6.46	2.88	14.50
	Motivational climate	1.14	14.91	<0.001	3.12	1.75	5.55
	Class management	0.97	10.16	0.001	2.64	1.45	4.80
Reference: LM/LP Target: HM/LP	Gender (male)	−0.14	0.44	0.508	0.87	0.57	1.33
	SEC	2.18	14.17	<0.001	8.87	2.85	27.61
	Specialized program	0.46	2.03	0.154	1.59	0.84	3.01
	Motivational climate	0.88	8.94	0.003	2.41	1.35	4.30
	Class management	0.88	8.42	0.004	2.42	1.33	4.39

HM/HP, high motivation and high performance; WM/EP, weak motivation with elevated performance; LM/LP, low motivation and low performance; HM/LP, high motivation with low performance; SEC, socio-economic–cultural background; 95% CI LB, lower bound of the 95% CI; 95% CI UB, upper bound of the 95% CI; and Exp(B), odds ratio.

## Discussion

The large variability of findings and, in some cases, weaker than expected associations may seem inconsistent with the view that achievement motivation is a key driver of performance at school, and vice versa. To further our understanding of the motivation–performance links, we analyzed the potential heterogeneity of the relationship between these constructs in two cohorts of middle school students. As expected, our findings revealed both concordant and discordant patterns of academic motivation and performance at school. Motivation and performance go hand in hand for some subgroups of students, while in other subgroups the levels of motivation and performance diverge. The subgroups also differ with regard to student socio-economic background, special educational needs, gender, well as perceptions of classroom climate. Below we discuss these findings in more detail.

### The Links Between Aspects of Motivation and Performance Among Middle School Students

Our study illustrates how the findings from variable- and person-oriented analyses provide a complementary view on the links between achievement motivation and performance at school. From a variable-oriented perspective, our findings suggest a weak to null association between motivation and performance at school, depending on the aspect of motivation and performance considered. In this respect, our findings are similar to, or somewhat lower than, the average estimates of the motivation–performance association reported in meta-analyses of studies with students and adult learners (Hansford and Hattie, 1982; Valentine et al., 2004; Huang, 2011; Bauer et al., 2015; Korpershoek et al., 2019). These low estimates of the links in focus may suggest that major aspects of achievement motivation, such as academic task value, academic self-concept, and school-related affect may be only marginally relevant for student performance at school, and vice versa. However, we argue that only examining the direct linear associations between aspects of achievement motivation and performance results in an incomplete understanding of these motivation–performance links. Indeed, it appears that the general academic functioning underlying the aspects of motivation and performance may be more complex than can be captured by linear, variable-oriented analyses. As a result, we suggest that using a person-oriented approach to study achievement motivation and performance enables us to obtain a more nuanced picture of how motivation and performance manifest among middle school students.

First of all, academic motivation covers a wide range of multifaceted motivational processes, which both co-vary and interact with learning processes, academic behaviors, and their outcomes (Vu et al., 2021). Thus, the relationships between specific aspects of motivation and performance may be not direct, but rather dependent on a complex interplay of different motivational processes (as illustrated by Putwain et al., 2019, as well as Marsh et al., 2005). Moreover, a direct estimate of an association on a sample level is an aggregate of the association in focus across different subgroups in a population, if such subgroups exist (Bergman et al., 2003). Thus, lower than expected or null correlations between theoretically related constructs may indicate certain heterogeneity in a population rather than the absence of any meaningful association between the constructs. Our findings based on a person-oriented approach suggest that this may be a more accurate description of the actual covariation of motivation and performance among middle school students.

Specifically, our findings revealed high levels of concordance between motivation and performance scores in two subgroups of 8th-grade students. In the subgroups characterized by *low motivation and low performance* (34% of the sample) and *high motivation and high performance* (18% of the sample) profiles, their levels of motivation went hand in hand with their scores on math and reading tests. This finding is in line with previous studies which identified large proportions of middle school students for whom the levels of motivation followed the same pattern as their academic performance. Indeed, three concordant profiles have been consistently reported in previous studies: one profile characterized by average performance and average motivation (68%, 41%, and 50% of the sample, respectively, in studies by Korhonen et al., 2014; Parhiala et al., 2018; Widlund et al., 2018), one with high performance and high motivation (11%, 34%, 25%, and 26% of the sample, respectively, in studies by Roeser et al., 1999; Korhonen et al., 2014; Parhiala et al., 2018; Widlund et al., 2018), and a profile with low levels of both motivation and performance (7%, 18%, and 23% of the sample, respectively, in Roeser et al., 1999; Korhonen et al., 2014; Parhiala et al., 2018).

In contrast, the other two profiles identified in our study had divergent patterns of motivation and performance. Specifically, the *weak motivation with elevated performance* profile (38% of all students) characterized a subgroup of students with lower than average motivation, but higher than average scores on math and reading tests. Similar profiles were also reported in some previous studies (Roeser et al., 1999; Korhonen et al., 2014). On the one hand, the salience of such subgroup suggests that students may demonstrate relatively high academic performance despite relatively low levels of achievement motivation (academic task value, academic self-concept, and positive school-related affect). On the other hand, EVT perspective suggests that some other aspects of achievement motivation, not assessed in our study, may be at play in this case.

Specifically, our study mostly focused on those aspects of achievement motivation, which are related to enjoyment, interest, and attainment (Eccles et al., 1983; Eccles and Wigfield, 2020). As conceptualized by Eccles and colleagues (Eccles et al., 1983; Eccles and Wigfield, 2020), these are the aspects of motivation that stem from within a person; they are either based on genuine pleasurable experiences in achievement-related settings, or on personal/identity-based importance attributed to academic tasks (Eccles and Wigfield, 2020). However, achievement motivation can also be supported by numerous external influences, beyond internal motivation, such as extrinsic rewards and requirements (Vu et al., 2021). In this case, either high utility of engaging in academic activities, or high cost of disengaging in them, may be at play in supporting students’ academic engagement and performance (Eccles and Wigfield, 2020). For example, a necessity to take graduation exams may facilitate engagement and performance because exam results may determine future educational and vocational possibilities of a student. Performance-oriented family discipline or peer norms may determine a high emotional cost of academic disengagement or low performance. Thus, students in *weak motivation with elevated performance* profile may rely on utility or cost considerations even in the absence of internal motives for studying, and this can still lead to relatively high performance. Performance can also improve due to efficient support for learning, such as high quality of teaching, educational resources, or study materials (Vu et al., 2021). Thus, achievement motivation of students in *weak motivation with elevated performance* profile may be dependent on either school, family, or peer structures that are favorable for good academic results and rich in educational resources. In line with this, a similar profile characterized by low academic value, but not reduced academic performance identified by Roeser et al. (1999) was related to a privileged social status, the largest family income, and highest parental educational attainment.

The second divergent profile was the *high motivation with low performance* group (9% of the sample). A similar profile was reported in a previous study with Finnish 9th-grade students (Widlund et al., 2018). Again, for these students their achievement levels must be determined by factors other than aspects of achievement motivation assessed in our study. For example, perceived utility of high academic performance may be low, or costs involved, such as effort, loss of valued alternatives, or emotional toll (Flake et al., 2015), needed to achieve high academic results may be perceived as too high. In addition, academic self-concept may be not congruent with actual performance due to factors as social comparisons or external feedback that are favorable despite low actual achievement levels (Vu et al., 2021). Large achievement gaps among schools in the Lithuanian education system (NEC, 2015, 2018) suggest that there may be schools and classrooms in which low levels of achievement on a population level would not be low compared to a classroom or school level.

In addition, certain conceptual attributes of global measures of motivation aspects, as measured in our study, may explain the profile with high motivation but low scores on math and reading tests. As summarized by Bong and Skaalvik (2003), academic self-concept in its broadest sense is based on social comparisons (e.g., within a classroom or peer group) rather than on objective performance standards. It is also based on cumulative past experiences rather than on present task-specific performance. Third, academic self-concept of a person may vary substantially across domains since appraisals can be made comparing one’s success in different domains (thus, a growing self-concept in one academic domain may lower self-concept in another domain). Fourth, due to a lack of objective criteria for success appraisals, academic self-concepts tend to cover the subjectively central aspects of an individual (but not necessarily reflect the essence of the academic domain or activity in question). All these attributes can explain why some students in our sample may be able to maintain high levels of motivation despite their objectively low levels of performance on math and reading, especially since performance was assessed with standardized tests rather than school grades, which may have stronger links to aspects of motivation (Korpershoek et al., 2019).

To summarize, for students in subgroups characterized by concordant profiles, the aspects of motivation assessed in our study (i.e., academic task value, academic self-concept, and school-related affect) are important factors behind their academic performance. However, for other students there is no such direct relationship between these motivational aspects and academic functioning. Our findings on the correlates of the profiles provide further insight into possible factors related to the specific patterns.

### Student and Classroom Characteristics Related to Profiles of Motivation and Performance

In line with our expectations, students from more favorable SEC backgrounds were more likely to show stronger motivation and performance. In fact, student socio-economic background differentiated between all identified profiles. Students with most favorable socio-economic backgrounds were most likely to belong to the *high motivation and high performance* profile, while those with the least favorable SEC background were most likely to belong to the *low motivation and low performance* profile. Importantly, when comparing this least favorable profile with the *weak motivation with elevated performance* profile, we can observe that despite very small differences in academic motivation between these profiles there are very large differences in the academic performance in the two subgroups, as well as moderate differences in special learning needs. We could think that the significant differences in socio-economic background and special learning needs, rather than motivation, are at the center of the gap in performance between these two profiles. Alternatively, based on EVT approach (Eccles et al., 1983; Eccles and Wigfield, 2020), we could argue that there are some utility or cost aspects of achievement motivation, closely related to low socio-economic background or high special learning needs, which impair academic engagement and performance of disadvantaged adolescent students.

While socio-economic background differentiated all four profiles (showing strong effects in all but one comparison), the other predictors showed somewhat weaker effects and a more specific pattern in profile comparisons. First, the proportion of students with special learning needs only differentiated the profiles with low vs. high performance (not with different motivation levels). In contrast, perceived motivational climate and classroom management differentiated between high vs. low motivation profiles, as in previous studies (Roeser et al., 1999), but not between those with different performance. Being male was weakly related to poorer motivation and performance.

Overall, profile comparisons suggest that the relationship between aspects of motivation and performance may be context-dependent. In contexts with high structural barriers, such as an unfavorable socio-economic background or salient special learning needs, some aspects of motivation may be at odds with performance. That is, high motivation may not lead to high performance due to inadequate structural conditions for learning. This observation is in line with previous meta-analytical findings that under the circumstances of unfavorable socio-economic conditions, the relationship between aspects of motivation and academic performance is less positive compared to favorable socio-economic contexts (Hansford and Hattie, 1982). Similarly, previous longitudinal findings revealed that among the students with advanced reading skills the links between academic motivation and reading performance is stronger compared to struggling readers (Klauda and Guthrie, 2015). Thus, cognitive challenges may also limit the relations of achievement motivation and performance. Another divergent pattern of motivation and performance may emerge in contexts where structural conditions support high performance, but students perceive classroom climate as unsupportive of their developmental needs. In such contexts, even students with relatively high performance may not have strong motivation to learn, but rather rely on external requirements and resources for academic performance.

Most importantly, profile comparisons also suggest that the four profiles may respond to completely different measures aimed at higher student performance at school. While students with high performance and reduced motivation may benefit from a more favorable motivational climate and more structure and predictability in a classroom, this would hardly be beneficial to students with high motivation and low performance. The latter profile may better respond to higher quality of learning resources and need-adjusted structural learning conditions at school. These insights need further exploration with longitudinal data on student performance, motivation, and learning context.

Finally, our findings revealed some country-specific variations in achievement motivation and performance among middle school students. Notably, the share of students characterized by profiles with low motivation in our study is substantially larger, while the share of those characterized by profiles with high levels of motivation is smaller than in previous person-oriented studies (Roeser et al., 1999; Korhonen et al., 2014; Parhiala et al., 2018; Widlund et al., 2018). Considering cross-national differences in the levels and gaps in academic performance, this is an expected finding. The results of both national and international studies on achievement indicate high proportions of low-performing students and large achievement gaps among Lithuanian students, classes, and schools, compared to high-performing countries with high levels of social equity in education, such as Finland (OECD, 2013, 2016, 2019; NEC, 2015, 2018). Taken together, these findings may indicate a low fit between adolescents’ salient developmental needs and the actual classroom environments in Lithuanian schools. Indeed, subgroups with low achievement motivation tended to perceive their classroom environment as less motivating, less structured, and less predictable, which may have undermined the aspects of their achievement motivation assessed in our study.

## Limitations and Future Directions

It is important that our findings were supported in two separate cohorts of 8th-grade students assessed with a 3-year break between them (2012 and 2015). However, more recent cohorts with larger breaks between assessments could be included in further person-oriented studies in order to see whether the various profiles of motivation and performance identified in our study persist. Moreover, longitudinal assessments of student cohorts could be utilized for this purpose, as demonstrated by Widlund et al. (2018).

With regard to the operationalization of motivational constructs, it is important to consider the multifaceted nature of achievement motivation, as conceptualized by EVT (Eccles et al., 1983; Eccles and Wigfield, 2020), and include diverse aspects of academic value, including utility value and different dimensions of cost (Flake et al., 2015). It may help to better explain the discordant patterns of achievement motivation and performance, which were identified in our study and previous person-oriented research in the field (Roeser et al., 1999; Korhonen et al., 2014; Parhiala et al., 2018; Widlund et al., 2018). It is also important to match subject areas between aspects of motivation and performance in future person-oriented studies in the field. Such an approach has consistently provided considerably stronger aggregate estimates of the motivation–performance link in variable-oriented studies compared to using global motivation measures (Valentine et al., 2004; Huang, 2011). Since most person-oriented studies to date have used global measures of motivational functioning at school, there is still a need to examine how typical patterns of subject-specific academic functioning may differ from the global patterns of motivation–performance.

Finally, our study was limited by the archival nature of the data used for the analyses. For example, students’ gender and residential location were assessed as binary variables, which does not reflect the actual variability and complexity of these characteristics.

## Data Availability Statement

Publicly available datasets were analyzed in this study. These data can be found at: https://www.nsa.smm.lt/stebesenos-ir-vertinimo-departamentas/tyrimai/nacionaliniai-tyrimai/nacionaliniai-mokiniu-pasiekimu-tyrimai-nmpt.

## Ethics Statement

Ethical review and approval was not required for the study on human participants in accordance with the local legislation and institutional requirements. Written informed consent from the participants' legal guardian/next of kin was not required to participate in this study in accordance with the national legislation and the institutional requirements. The data used in this study were collected by the national authorities according to the national legal regulations of Lithuania (Regulations for conducting studies of student achievement, 2008), which include provisions on ethics and confidentiality of the study participants.

## Author Contributions

RE conceived of the study, contributed to statistical analyses, and drafted the manuscript. RV supervised and performed statistical analysis and contributed to drafting the manuscript. DS performed data cleaning and preparation and contributed to data analyses. EM and SR contributed to drafting the manuscript. DD provided feedback on statistical analyses and contributed to drafting the manuscript. All authors contributed to the article and approved the submitted version.

## Funding

This study has received funding from European Regional Development Fund (project no 01.2.2-LMT-K-718-03-0059) under grant agreement with the Research Council of Lithuania (LMTLT) and by a grant from the Wenner-Gren Foundations (GFOh2020-0003).

## Conflict of Interest

The authors declare that the research was conducted in the absence of any commercial or financial relationships that could be construed as a potential conflict of interest.

## Publisher’s Note

All claims expressed in this article are solely those of the authors and do not necessarily represent those of their affiliated organizations, or those of the publisher, the editors and the reviewers. Any product that may be evaluated in this article, or claim that may be made by its manufacturer, is not guaranteed or endorsed by the publisher.

## Footnotes

^1^ https://www.nsa.smm.lt/stebesenos-ir-vertinimo-departamentas/tyrimai/nacionaliniai-tyrimai/nacionaliniai-mokiniu-pasiekimu-tyrimai-nmpt/

## Appendix

**ANNEX 1** Items used to assess students’ academic motivation and teaching characteristics, and the internal consistency of the scales.

**Table:**

**Concept**	**Items in Lithuanian**	**Items in English**	**2012 Cronbach’s *α***	**2015 Cronbach’s *α***
Academic task value	Tau yra svarbu gerai mokytis	It is important for you to study well	0.77	0.79
	Mokaisi noriai	You study willingly		
	Jautiesi atsakingas už savo mokymąsi	You feel responsible for your own learning		
	Žinai, ko Tau svarbu išmokti	You know what you need to learn		
	Tau svarbu suprasti dalyko mokymosi tikslus	It is important for you to understand the learning objectives of the subject		
Academic self-concept	Nebijai mokymosi sunkumų	You are not afraid of learning challenges	0.69	0.78
	Mokydamasis pasitiki savo jėgomis	You trust in yourself when learning		
	Žinai įvairių būdų, padedančių atlikti ne tik tas užduotis, kurios Tau patinka, bet ir tas, kurios sunkios ar nuobodžios	You know different ways to accomplish both the tasks you like and the ones that are difficult or boring		
	Tau gerai sekasi sutelkti dėmesį mokantis	You are good at focusing on learning		
School-related affect	Mokykloje Tu jautiesi saugus	You feel safe at school	0.72	0.76
	Tau patinka būti mokykloje	You love being at school		
	Tau patinka mokytis savo mokykloje	You enjoy studying at your school		
	Klasėje jautiesi gerai	You feel good in class		
Motivational climate	Mokytojai pasirūpina, kad mums būtų įdomu mokytis	Teachers make sure we are interested in learning	0.76	0.79
	Mokytojai Tau padeda suprasti, ko ir kodėl mokaisi	Teachers help you understand what you are learning and why		
	Mokytojai mus moko mokytis įvairiais būdais	Teachers teach us to learn in a variety of ways		
Classroom management	Su mokytojais aptariame, kaip atliksime vieną ar kitą užduotį, kiek tam reikės laiko ir pan.	We discuss with teachers how we will complete one task or another, how long it will take, and so on	0.82	0.85
	Mokytojai mums pataria, kaip lengviau būtų išmokti vieną ar kitą dalyką	Teachers advise us how to easier to learn one thing or another		
	Mokytojai paaiškina, su kokiais sunkumais galime susidurti atlikdami užduotį ir ką reikėtų tada daryti	Teachers explain what difficulties we may face in completing the task and what should be done then		
	Kartu su mokytojais išbandome įvairius būdus, kurie padeda mums stebėti savo mokymosi pažangą	Together with teachers, we try out different ways that help us track our learning progress		
	Mokytojai pataria ir stebi, kad darbą atliktume laiku	Teachers advise and monitor that we get the job done on time		
	Pamokose atliekant sunkias užduotis mokytojai pasiūlo keletą minučių pailsėti (poilsio pertraukėlių)	In difficult tasks, teachers offer a few minutes of rest (rest breaks)		
	Mokytojai suteikia Tau reikalingą pagalbą, kad neleistum mokymosi laiko veltui	Teachers give you the help you need not to waist your learning time		
Teacher-student relationship	Mokytojai palaiko mus, skatina pasitikėti savo jėgomis	Teachers support us, encourage us to trust in our own strengths	0.79	0.80
	Mokytojai pastebi ir paskatina mūsų pastangas mokytis	Teachers notice and encourage our efforts to learn		
	Mokytojai skiria laiko pasikalbėti su Tavimi apie tai, kaip Tau sekasi mokytis	Teachers take the time to talk to you about how you are doing		
	Mokytojai pasako Tau, ką atlieki gerai, ir pataria, kaip galėtum pasiekti geresnių rezultatų	Teachers tell you what you are doing well and advise you on how you can achieve better results		
